# Respiratory Virus-Specific and Time-Dependent Interference of Adenovirus Type 2, SARS-CoV-2 and Influenza Virus H1N1pdm09 During Viral Dual Co-Infection and Superinfection In Vitro

**DOI:** 10.3390/v16121947

**Published:** 2024-12-19

**Authors:** Maria Alfreda Stincarelli, Rosaria Arvia, Bernardo Guidotti, Simone Giannecchini

**Affiliations:** Department of Experimental and Clinical Medicine, University of Florence, Viale Morgagni 48, I-50134 Florence, Italy; mariastincarelli@gmail.com (M.A.S.); rosaria.arvia@unifi.it (R.A.); bernardo.guidotti@edu.unifi.it (B.G.)

**Keywords:** influenza virus, SARS-CoV-2, adenovirus, co-infection, superinfection

## Abstract

Background. Understanding the interference patterns of respiratory viruses could be important for shedding light on potential strategies to combat these human infectious agents. Objective. To investigate the possible interactions between adenovirus type 2 (AdV2), severe acute respiratory syndrome coronavirus 2 (SARS-CoV-2) and influenza A/H1N1 pandemic (H1N1pdm09) using the A549 cell line. Methods. Single infections, co-infections, and superinfections (at 3 and 24 h after the first virus infection) were performed by varying the multiplicity of infection (MOI). Virus replication kinetics and the mRNA expression of IFN-α, IL-1α and IL-6 were assessed by real-time qPCR. Results. Co-infection experiments showed different growth dynamics, depending on the presence of the specific virus and time. AdV2 replication remained stable or possibly enhanced in the presence of co-infection with each of the two H1N1pdm09 and SARS-CoV-2 viruses used. In contrast, SARS-CoV-2 replication was facilitated by H1N1pdm09 but hindered by AdV2, indicating possible different interactions. Finally, H1N1pdm09 replication exhibited variably effectiveness in the presence of AdV2 and SARS-CoV-2. Superinfection experiments showed that the replication of all viruses was affected by time and MOI. The mRNA expression of IFN-α, IL-1α and IL-6 showed divergent results depending on the virus used and the time of infection. Conclusions. Further investigation of co-infection or superinfection may be helpful in understanding the potential relationship involved in the outcome of viral respiratory infection in the human population.

## 1. Introduction

Respiratory viruses are common pathogens responsible for millions of illnesses worldwide each year [1,2]. The seasonality of respiratory virus circulation (which includes enveloped and naked viruses with DNA and RNA genomes) is well known. Epidemics of influenza viruses, human coronaviruses and human respiratory syncytial virus (HRSV) occur in winter; some enteroviruses occur in summer, and adenoviruses, human bocaviruses, human metapneumoviruses and rhinoviruses occur throughout the year with parainfluenza viruses possessing type-specific pattern of seasonal circulation [1]. Due to its high infection and mortality rates, many public health strategies have been used to control the spread of the disease [1]. These include vaccination, social and physical isolation, and restriction of international travel [1]. The co-infection and superinfection of respiratory viruses have also been found to be associated with the worsening of host disease [3]. Simultaneous infection with more than one respiratory virus has been a focus in the study of viral infection interactions [4,5,6,7,8]. Co-infection is the simultaneous infection of a host with at least two pathogens, which usually leads to increased disease severity, diagnostic difficulties, and stress on the immune response [3,9]. Superinfection occurs when a second infection combined with a first one is ongoing, resulting in severe clinical manifestations and sometimes complicating the disease management [10,11,12,13]. Interactions between respiratory viruses in co-infection and superinfection can affect disease severity and influence diagnosis, management, and treatment [14,15,16,17]. Respiratory viruses induce a strong immune response mainly by stimulating cells to produce cytokines, which are also involved in the regulation of the immune response against respiratory viruses [18,19]. Of all the cytokines involved, interferon-alpha (IFN-α), interleukin 1 alpha (IL-1 α) and interleukin-6 (IL-6) are of particular interest in this type of infection [20,21,22]. Because of the persistent emergence of new respiratory virus strains, the scenario of co-infection and superinfection is still relevant for diseases and viruses [23,24,25]. Therefore, studies are needed to understand the potential positive or negative interaction of different respiratory viruses and the role of cytokines during co-infection and superinfection. Here, among the respiratory viruses relevant to human health, it was found interesting to investigate the effect of co-infection and superinfection of influenza viruses, adenovirus (AdV) and severe acute respiratory syndrome coronavirus 2 (SARS-CoV-2). In particular, influenza viruses are enveloped viruses with a negative single-stranded segmented RNA genome belonging to the family *Orthomyxoviridae*. They can cause seasonal influenza with mild to severe symptoms and trigger pandemics that challenge global health systems [26,27]. AdVs, double-stranded naked DNA viruses, belong to the family *Adenoviridae* and are responsible for a wide range of diseases including respiratory, gastrointestinal and ocular infections [28]. While most adenoviral infections are mild, some types, like AdV2, can cause severe respiratory disease, particularly in children and the immunocompromised [29]. These infections can cause severe manifestations such as pneumonia, bronchiolitis and acute respiratory distress syndrome (ARDS) [30]. SARS-CoV-2, a positive-sense, single-stranded enveloped RNA virus, is a new emerging virus of the *Coronaviridae* family that caused the COVID-19 pandemic in 2020 [31]. It can cause a range of respiratory infections, from asymptomatic to severe pneumonia [32,33]. Thus, the co-infection and superinfection of influenza virus A/H1N1pdm09, AdV2 and SARS-CoV-2 were performed in an A549 cell substrate monitoring viral replication at different points of infection. Moreover, the effect of IL1α, IL-6 and IFN-α cytokine mRNA expression was also investigated.

## 2. Materials and Methods

### 2.1. Cells and Viruses

A549 (adenocarcinomic human alveolar basal epithelial cells, ATCC CCL-185) was cultivated using Dulbecco’s Modified Eagle Medium (DMEM, Merck, Darmstadt, Germany) culture medium supplemented with 10% fetal bovine serum (FBS). The viruses used were SARS-CoV-2 clinical isolate (SCV2/FI/2/21 Delta-like variant); influenza virus A/Firenze/02/2019 H1N1pmd09 strain and AdV2 grown on A549 cells and titrated by the plaque method. The viral stocks, consisting of cell-free supernatants of acutely infected cells, were aliquoted and stored at −80 °C until used.

### 2.2. Infection Experiments

#### 2.2.1. Co-Infections

Twenty-four hours prior to infection, A549 cells were seeded in 24-well plates at a density of 2.5 × 10^4^ cells per well and incubated overnight at 37 °C in a 5% CO_2_ atmosphere to allow adhesion and reach approximately 80% confluence. The next day, the cells were simultaneously infected with two viruses used at different combinations of multiplicity of infection (MOI) of 0.1, 0.01 and 0.001. As a control of viral replication, cells infected with each of the individual viruses under the same conditions were used. The inoculum used was prepared by diluting the viral stock in 100 μL of serum-free DMEM and added to the cells. The cells were incubated for 1 h at 37 °C; then, the inoculum was removed and the cells were washed with PBS to remove unbound virus. New serum-free DMEM was added to the cells; then, they were incubated at 37 °C in a 5% CO_2_ atmosphere. The supernatants and cells were collected 3, 24, 48, 72 h after infection (hpi) and stored at −80 °C until nucleic acid extraction.

#### 2.2.2. Superinfections

Twenty-four hours prior to infection, A549 cells were seeded in 24-well plates at a density of 2.5 × 10^4^ cells per well and incubated overnight at 37 °C in a 5% CO_2_ atmosphere to allow adhesion and reach approximately 80% confluence. The following day, cells were infected with the first virus inoculum prepared in 100 μL of serum-free DMEM at a multiplicity of infection (MOI) of 0.01. The cells were incubated for 1 h at 37 °C; then, the inoculum was removed and the cells were washed with PBS to remove unbound virus. Then, fresh serum-free DMEM was added to the cells, which were subsequently incubated at 37 °C in a 5% CO_2_ atmosphere. After 3 and 24 h, the cells were superinfected with the second virus inoculum, prepared under the same conditions as described for the first virus inoculum, at different MOI values of 0.1, 0.01 and 0.001. As a control of viral replication, cells infected with the first virus inoculum were treated in a simulated manner with serum-free DMEM at the same time and under the same conditions used for the superinfection. In addition, as a control for the second infection, cells were mock-treated with serum-free DMEM for 3 and 24 h prior to infection with the second virus used in the superinfection. Supernatants and cells were collected 3, 24, 48 and 72 h after infection (hpi) and stored at −80 °C until extraction of the nucleic acid was performed.

### 2.3. RNA Extraction, Reverse Transcription and Real-Time PCR

RNA was extracted from 150 μL of supernatant with the QIAamp RNA mini Kit (Qiagen, Hilden, Germany) and from infected cells (1 × 10^5^) with the RNAeasy mini kit (Qiagen) according to the manufacturer’s instructions. One hundred nanograms of total RNA of each sample were reverse-transcribed and amplified by real-time PCR in a 25 μL PCR reaction mix (AgPath-ID One-Step RT-PCR, Applied Biosystems, Waltham, MA, USA) using primers targeting the N gene of SARS-CoV-2 (primer forward SC2-For 5′ CTG CAG ATT TGG ATG ATT TCT CC 3′, reverse SC2-Rev 5′ CCT TGT GTG GTC TGC ATG AGT TTA G 3′ and probe SC2-Probe FAM-5′ ATT GCA ACA ATC CAT GAG CAG TGC TGA 3′-MGB) and using primers targeting the M gene of the H1N1pdm09 Influenza A virus (primer forward InfA-For 5′ GAC CRA TCC TGT CAC CTC TGAC 3′reverse, InfA-Rev 5′ AGG GCA TTY TGG ACA AAK CGT CTA 3′ and probe InfA-prob FAM– 5′ TGC AGT CCT CGC TCA CTG GGC ACG 3′-MGB). The mix was amplified in a Rotor-Gene Q real-time instrument (Qiagen), and the reaction was carried out at 95 °C for 10 min followed by 40 cycles (20 s at 95 °C, 60 s at 60 °C; the fluorescence was recorded at 70 °C). All reactions were performed in duplicate.

### 2.4. DNA Extraction and Real-Time PCR

Viral DNA was extracted from 200 μL supernatant and from infected cells (1 × 10^5^) using an QIAmp DNA mini Kit (Qiagen) according to the manufacturer’s instructions. One hundred nanograms of total DNA of each sample were amplified by real-time PCR in a 20 μL PCR reaction mix (SsoAdvanced Universal SYBR Green Supermix, Bio-Rad, Hercules, CA, USA) using primers targeting the region of the hexon gene of AdV2 (primer forward 5′ CCC ITT YAA CCA CCA CCG 3′, reverse 5′ ACA TCC TTB CKG AAG TTC CA). The mix was amplified in a Rotor-Gene Q real-time instrument (Qiagen), and the reaction was carried out at 95 °C for 2 min, which was followed by 40 cycles (15 s at 95 °C, 30 s at 60 °C); the fluorescence was recorded using the Melt-Curve analysis 2 s/step 65–95 °C with a 0.5 °C increment. All reactions were performed in duplicate.

### 2.5. Cytokine mRNA Expression Studies

At 3 and 24 h post-infection or superinfection, A549 were analyzed to study the mRNA expression of IL-1α, IL-6 and IFN-α using validated PrimePCR SYBR Green Assays (Bio-Rad, Hercules, CA, USA). Comparative RT-PCRs were carried out using SsoAdvanced Universal SYBR Green Supermix (Bio-Rad, Hercules, CA, USA). Expression of the selected genes in three independent infected cultures was displayed as fold change compared to mock-infected cultures and cells infected with only one virus using the ΔΔCt method. The expression of target genes was normalized to the expression of the 18S gene.

### 2.6. Statistical Analysis

Statistical analyses were performed using the one-way ANOVA with Bonferroni’s correction test. All values are showed as means ± standard deviation. Moreover, data were analyzed using a two-tailed Student’s *t*-test. Associations between variables were determined by applying the Pearson’s correlation coefficient. Differences were considered statistically significant when *p* < 0.05. Statistical analyses were conducted using the GraphPad Prism software.

## 3. Results

### 3.1. Kinetic of Virus Replication in Co-Infection and Superinfection

To analyze virus growth during co-infection and superinfection, virus infection experiments were performed at different MOIs examining virus replication at different post-infection times. First, it was observed that the three viruses in single infection produced an infection titer of 50% of the tissue culture infectious doses of 10^6^/mL, 5.4 × 10^5^/mL and 1.5 × 10^5^/mL for AdV2, H1N1pdm09 and SARS-CoV-2, respectively. Furthermore, all three viruses produced a clear cytopathic effect in the A549 cell line in all single infections.

**Figure 1:**
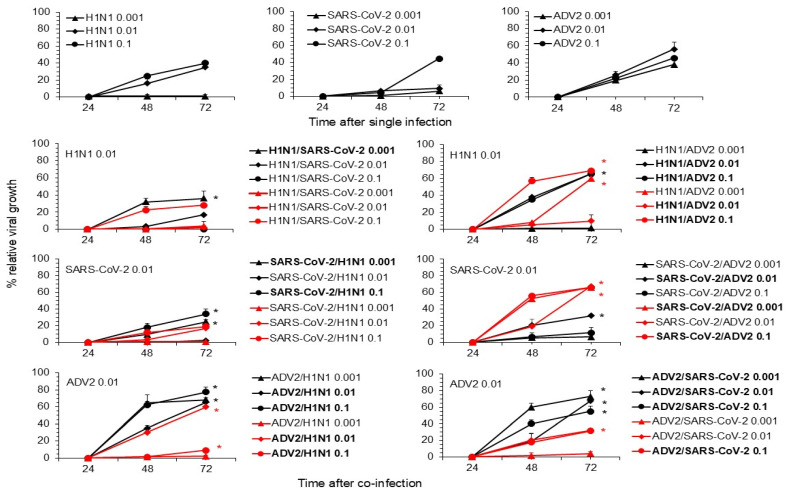
Effect of co-infection on virus replication. A549 cells were co-infected with the combination of the two indicated viruses at multiplicity of infection (MOI) values of 0.1, 0.01 and 0.001. The upper panels report each individual viral replication at the indicated MOI. The lower panels report the viral replication of the indicate virus used at a fixed MOI of 0.01 in the presence of co-infection with one of the other viruses at three different MOIs (lower, equal and higher). The supernatant of the infected cells was collected after 24, 48 and 72 h post-infection (hpi) and used to extract RNA or DNA. One hundred nanograms of total RNA or DNA were amplified using primers and probes specific for AdV2, H1N1pdm09 and the genomic region of SARS-CoV-2, as reported in the Section 2. Amplification of a specific viral target in the co-infection is indicated with a different color (black and red) and using the same symbol as the related virus in the single infection. The kinetics of viral growth was obtained by comparing the ct values of each virus used in the co-infection with the ct values of the same virus used in the single infection at each time following infection. The values reported are the mean + standard deviation obtained in 3 independent experiments. Virus co-infection with significant differences from the single infection is highlighted in bold. * *p* < 0.05, Student’s *t*-test).

Then, in co-infection and superinfection, each of the three selected viruses was used at a fixed infectious dose in the presence of a different infectious dose (lower, equal and higher) of one of the other viruses. Under co-infection conditions, as shown in Figure 1, the co-infection of AdV2 with one of the other two viruses generally results in higher viral replication kinetics than single infection. At the selected time points examined, the reduction in cell viability and the cytopathic effect were not significantly different between co-infection and single infection. Of note, the replication of AdV2 was enhanced at all infectious doses during co-infection with the other two viruses, showing mainly dose-dependent replication kinetics (Figure 1). SARS-CoV-2 showed variable replication patterns during co-infection with H1N1pdm09 and AdV2. When cells were co-infected with H1N1pdm09, SARS-CoV-2 replication increased, suggesting a beneficial effect determined by the interaction between these two RNA viruses. In contrast, co-infection with AdV2 had a mainly negative effect on SARS-CoV-2 replication (Figure 1). Finally, H1N1pdm09 showed variability in the increase in its replication activity when co-infected with AdV2 or SARS-CoV-2.

**Figure 2:**
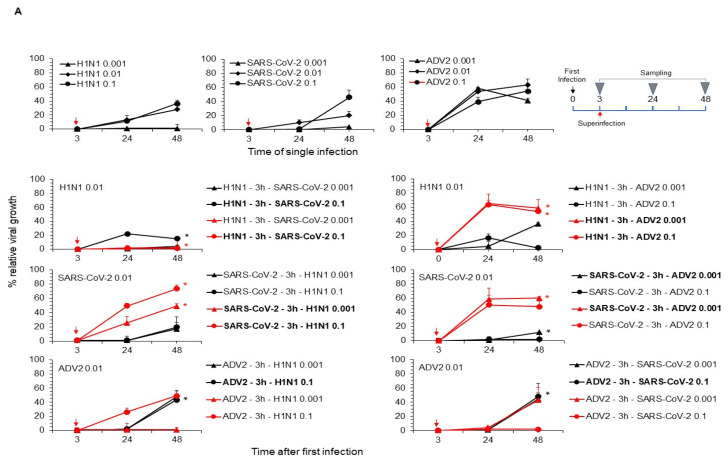

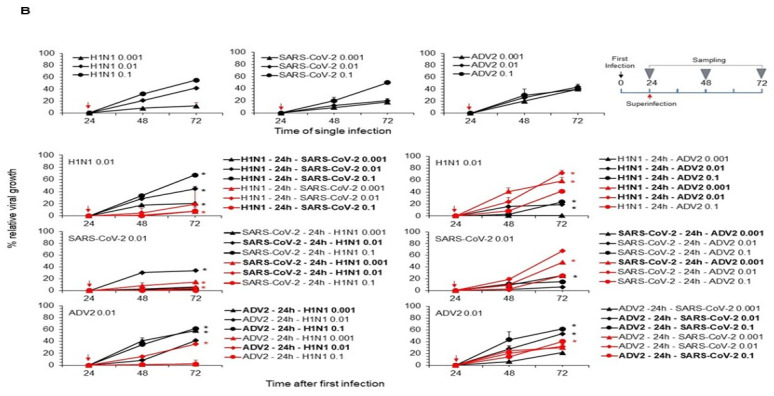
Effect of superinfection on viral replication. A549 cells were infected with a first virus at a multiplicity of infection (MOI) of 0.01. Then, after 3 hpi (**A**) or 24 hpi (**B**), a second infection was performed with viral inoculum at three indicated MOIs and prepared under the same conditions as described for the first infection. In (**A**,**B**), the upper panels show each individual virus replication at the indicated MOI, which was performed as described in the Section 2. In addition, a schematic of the superinfection virus addition and temporal sampling is shown. The lower panels report viral replication of the indicated virus used at a fixed MOI of 0.01 in the presence of co-infection with one of the other viruses performed at three different MOIs (lower, equal and higher). The supernatant of the infected cells was collected at 3, 24 and 48 hpi after infection (panel **A**, superinfection performed at 3 hpi after initial infection) or 24, 48 and 72 hpi after infection (panel **B**, superinfection performed at 24 hpi after initial infection) and used for RNA or DNA extraction. One hundred nanograms of total RNA or DNA was amplified using primers and probes specific for AdV2, H1N1pdm09 and the genomic region of SARS-CoV-2 as reported in the Section 2. Amplification of a specific viral target in the superinfection is shown in different colors (black and red) and with the same symbol as the corresponding virus in the song. The kinetic of viral growth was obtained comparing ct values of each virus used in superinfection to ct values of the same virus used in single infection at each time post-infection. Values shown are mean + standard deviation obtained in 3 independent experiments. Virus co-infection with significant difference compared to single infection is highlighted in bold. * *p* < 0.05, Student’s *t*-test.

Superinfection experiments were then performed. Again, a fixed infectious dose of the first virus was used, followed by different infectious doses (lower, equal and upper) of the second virus, which were added 3 h or 24 h after the first infection. In superinfection experiments, the timing and order of specific viral infections were critical factors influencing viral replication processes. As in the co-infection experiment, reduced cell viability and cytopathic effect did not differ significantly between superinfection and single infection at the selected time points examined. Notably, AdV2 replication was reduced in SARS-CoV-2 or H1N1pdm09 virus superinfected at 3 h after initial infection, whereas it was increased in these viruses superinfected at 24 h after initial infection (Figure 2A,B). Of note, in the latter superinfection at 24 h, the second virus showed reduced replicative activity compared to the single infection. Furthermore, these effects were dose dependent on the second virus used. The replication pattern of SARS-CoV-2 and H1N1pdm09 during superinfection was found to be time-dependent and virus-specific as well as dependent on the infectious dose used. The replication kinetics of H1N1pdm09 or SARS-CoV2 in superinfection with the other two viruses at 3 hpi was marginally reduced or similar compared to the single infection. In this experiment, the second virus used in superinfection exhibited mainly an increased replication. Of note, in the superinfection at 24 hpi of H1N1pdm09 with each of the other viruses, their replicative activity was associated with increased or decreased activity compared to the single infection (Figure 2A,B). Conversely, in the superinfection at 24 hpi of SARS-CoV-2 with each of the other viruses, its replicative activity decreased compared to that with a single infection (Figure 2B).

### 3.2. Cytokine mRNA Expression Profiles During Co-Infection and Superinfection

**Figure 3:**
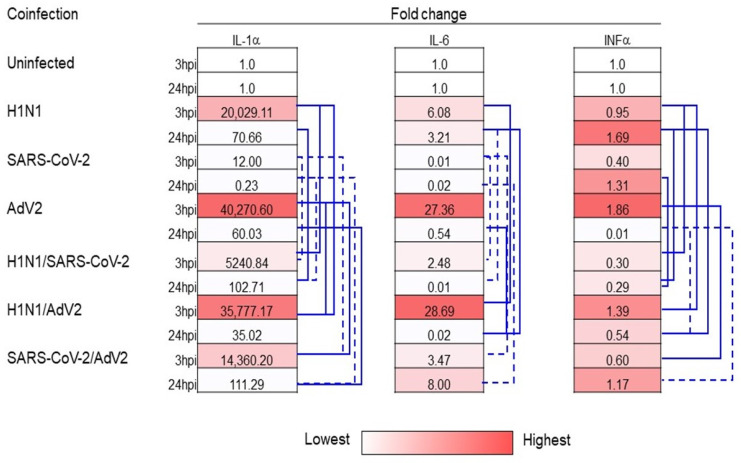
Expression of cellular innate immune response gene transcription during co-infection at different endpoints. A549 cells were co-infected with the combination of the two indicated viruses at a multiplicity of infection (MOI) of 0.01. The infected cells were collected 3 and 24 h after infection and used to extract RNA. One hundred nanograms of total RNA were amplified using primers and probes specific for interleukin-1 alpha (IL-1α), interferon-alpha (IFN-α) and interleukin-6 (IL-6). The mRNA expression levels of interleukin-1 (IL-1α), interferon-alpha (IFN-α) and interleukin-6 (IL-6) as indicators of cellular innate immune response were measured at 3 and 24 h after infection. The expression of selected genes in three independent co-infected cultures was visualized as a fold change compared with sham-infected cultures and cells infected with a single virus using the ΔΔCt method. The mRNA expression of target genes was normalized to 18S gene expression. Values reported are the average obtained in 3 independent experiments. Cytokine mRNA expression between co-infection and single infection with significant differences (solid line *p* < 0.05, dashed line *p* < 0.01, Student’s *t*-test) is reported.

Respiratory virus infection elicits a strong immune response mostly through the stimulation of cells to produce cytokines [18,19]. Of all the cytokines involved, interferon-alpha (IFN-α), interleukin-1α (IL-1α) and interleukin-6 (IL-6) are of particular interest in this type of infection [20,21,22]. Thus, IL-1α, IFN-α and IL-6 mRNA expression were measured at 3 hpi and at 24 hpi in co-infection (Figure 3).

Evaluating single infection, all three cytokines were highly expressed during AdV2 compared to H1N1pdm09 and SARS-CoV-2 especially when they were analyzed at 3 hpi. Infection with AdV2 showed a reduction in expression for IL-6 and INFα at 24 hpi compared to the uninfected control cells. Infection with SARS-CoV-2 showed a decreased expression of IL-6 measured at 3 and 24 hpi and of IFNα at 3 hpi and IL-1α at 24 hpi compared to uninfected control cells. All other infections caused a positive increase in the expression of the three cytokines compared to uninfected control cells. Analyzing the co-infection expression profile, the co-infection of AdV2 with H1N1pdm09 or SARS-CoV-2 resulted in lower levels of almost all three cytokines than AdV2 alone at 3 hpi. On the other hand, cytokine expression at 24 hpi of co-infection with AdV2 was lower for all three cytokines during co-infection with SARS-CoV-2 and IFNα with H1N1pdm09. In contrast, SARS-CoV-2 co-infection, except for INFα at 24 hpi, showed higher levels for the three cytokines in the presence of AdV2 and H1N1pdm09 compared to its single infection at both time points. H1N1pdm09 showed different efficacy during co-infection compared to single infection, depending on the virus used and the cytokine tested at 3 hpi.

**Figure 4:**
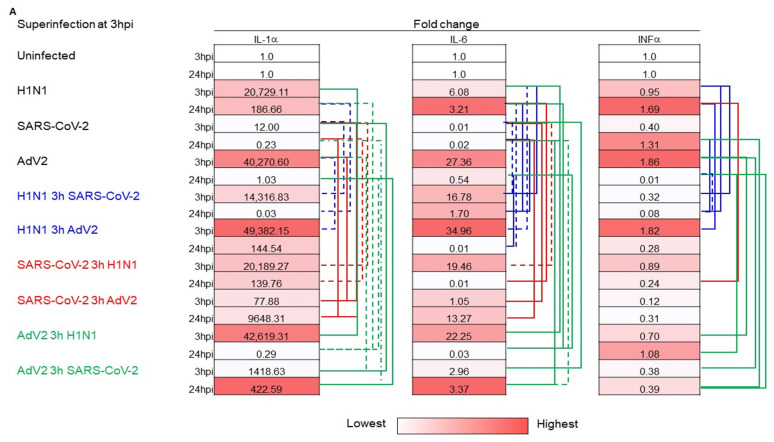

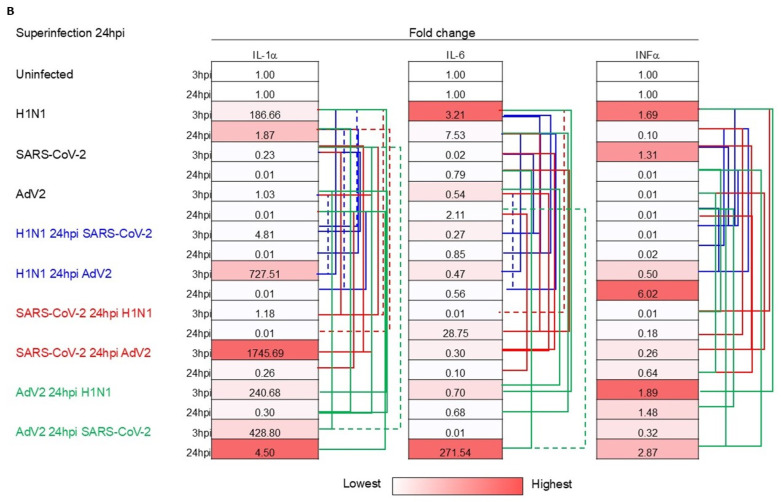
Expression of cellular innate immune response gene transcription during superinfection at different endpoints. A549 cells were infected with a first virus at a multiplicity of infection (MOI) of 0.01. Then, after 3 or 24 h, the second infection was performed at a multiplicity of infection (MOI) of 0.01. Superinfection experiments performed at 3 hpi (**A**) and 24 hpi (**B**) with different combinations of H1N1, SARS-CoV-2 and AdV2 are reported. Infected cells were collected after 3 and 24 hpi and used to extract RNA. One hundred nanograms of total RNA were amplified using primers and probes specific for interleukin-1 (IL-1α), interferon-alpha (IFN-α), and interleukin-6 (IL-6) The mRNA expression levels of interleukin-1 (IL-1α), interferon-alpha (IFN-α), and interleukin-6 (IL-6) were measured at 3 and 24 hpi after the first infection. The expression of selected genes in three independent superinfected cultures was visualized as fold change compared with mock-infected cultures and cells infected with a single virus using the ΔΔCt method. Target gene expression was normalized to 18S gene expression. Values reported are the average obtained in 3 independent experiments. Cytokine mRNA expression between superinfection and single infection with significant differences (solid line *p* < 0.05, dashed line *p* < 0.01, Student’s *t*-test) is reported.

In particular, IL-1α was decreased in co-infection with SARS-CoV-2 and increased with AdV2. IL-6 and IFNα showed contrasting expression depending on co-infection with SARS-CoV-2 or AdV2. On the other hand, the co-infection of H1N1pdm09 with SARS-CoV-2 or AdV2 resulted in lower levels of almost all three cytokines when tested at 24 hpi. During superinfection experiments, the different cytokine expression was dependent on the order of the virus used in infections from the interval time between the two infection and the time where cytokines were measured (Figure 4A,B). When superinfection was performed 3 h after the first infection and cytokine expressions were checked 3 h after the second infection, the superinfection of AdV2 with the addition of H1N1pdm09 or SARS-CoV-2 and vice versa resulted in lower expression levels than those obtained in single infection (Figure 4A,B). In contrast, infection with SARS-CoV-2 and superinfection after 3 h with AdV2 or H1N1pdm09 showed higher expression of the three cytokines than in the case of co-infection. Infection with H1N1pdm09 showed a higher expression of all three cytokines during superinfection with AdV2 and mainly lower expression with SARS-CoV-2 than those obtained in its single infection analyzed at 3 hpi. When cytokine expression was assessed at 24 hpi, the sequence of the virus type used in the infection gave mixed results. The effect on cytokine expression depended on the type of virus and the different superinfection times used. In the superinfection experiment, the expression of the three cytokines differed from that observed at 3 hpi, particularly for AdV2 superinfected at 24 hpi with SARS-CoV-2 or H1N1pdm09 (Figure 4A,B).

Overall, Table 1 shows that changes in viral replication during co-infection and superinfection sometimes significantly correlate with the fold change in cytokine expression during dual infection.

## 4. Discussion

This study describes the kinetics of viral replication of AdV2, SARS-CoV-2 and H1N1pdm09 during their dual co-infection and superinfection of the A549 cell line. Overall, it was observed that during co-infection, the H1N1pdm09 and SARS-CoV-2 viruses exerted an increased activity on the replication of the other virus. The superinfection experiments showed that the viral replication was dependent on the time of introduction of the superinfecting virus and on the infectious doses that were used. Of particular interest, AdV2 replication was reduced 3 h after superinfection with each of the other two viruses. In contrast, 24 h after superinfection with the same viruses, AdV2 replication was higher than in the single infection. Notably, the replication activity of the first virus was inversely related to the replication activity of the superinfecting virus, indicating a potential opposing interaction between the two superinfecting viruses. In addition, a reduced level of mRNA expression of the cytokines IL-1α, IL-6 and IFNα was observed during co-infection compared to single infection. Furthermore, in the superinfection performed 3 and 24 h after the first viral infection, the change in viral replication was sometimes found to correlate with the change in cytokines observed in the double infection. To date, several studies have reported that co-infection with respiratory viruses is recurrent for some specific viruses such as influenza virus, SARS-CoV-2 and respiratory syncytial virus [34,35,36]. However, co-infection of these viruses with other respiratory viruses may be rare [37,38,39]. In vivo studies have not always reported increased replication or worsened infection outcome during co-infection with SARS-CoV2 and H1N1pdm09 [40,41]. Furthermore, few studies have reported co-infection of SARS-CoV-2 or H1N1pdm09 with AdV2 in vivo [42,43]. In this context, there are few studies on the effect of SARS-CoV-2, H1N1pdm09 and AdV2 in co-infection or superinfection in vitro. Generally, it has been reported that during co-infection, two viruses can have a positive interaction and cooperation to reduce the host response [44,45]. However, the mechanism by which co-infection increases disease severity is poorly understood. It is possible that viral synergism promotes immune activation and immunopathology [46]. The role of modulation of the host immune response, promoting viral escape from antiviral activity, exerted by influenza virus and SARS-CoV-2 is well known [35]. Both viruses, like other RNA genome viruses, have a high capacity to modulate IFNα, which is one of the key players in the antiviral state [47]. Moreover, it has also been seen that IL-1α, a player of inflammatory response [48] and IL-6, an interleukin involved in immune responses, inflammation and hematopoiesis, have been associated with viral infections such as SARS-CoV-2 and influenza virus [49,50,51]. Thus, in our study, modulation of the mRNA expression of the cytokines IL-1α, IL-6 and IFNα by H1N1pdm09 or SARS-CoV-2 may have promoted enhanced viral replication during co-infection. However, an additional effect of other cytokines or a direct interaction with the virus in the modulation of virus replication during superinfection cannot be excluded. In vivo investigations reporting a worsening of AdV2 infection in the presence of H1N1pdm09 or SARS-CoV-2 confirm their activity in enhancing viral replication during co-infection [51]. In contrast, as observed in our study, during superinfection carried out at different times, the efficacy of these viruses may be opposite or unable to promote the replication of a first viral infection. The latter suggestion may account for some conflicting results of co-infection studies of these respiratory viruses in patients [46,50,51].

Therefore, the increased or decreased replication and worse disease severity observed with the co-infection of viruses in vivo may depend on the time of infection. In this context, the positive or negative interactions of respiratory RNA or DNA viruses may be related to several factors. These include their different time-dependent abilities to modulate cytokine response during infection, molecular characteristics, and use of the same or different intracellular localization of viral replication machinery [21,52,53]. In conclusion, co-infection and superinfection by respiratory viruses should be thoroughly investigated to better explain the potential impact on disease outcome in humans.

## Figures and Tables

**Table 1:** Overall effect of co-infection and superinfection on viral replication and cytokine mRNAS expression.

Type of Infection	Effect on Virus Replication ^a^	Effect on Fold Changes ^b^		
		IL-1α	IL-6	INFα
Co-Infection				
H1N1-SARS-CoV-2	H1N1 ↑/SARS-CoV-2↑	↓/↑	↓/↑	↓/None
H1N1-AdV2	H1N1 ↑/AdV2 ↑	↑/↓	↑/None	↑/↓
AdV2-SARS-CoV-2	AdV2 ↑/SARS-CoV-2↓	↓/↑(−0.95 *p* = 0.047)	↓ (−0.97 *p* = 0.003)/↑ (−0.95 *p* = 0.047)	↓ (−0.96 *p* = 0.003)/↑
Superinfection at 3 h				
H1N1 3 h SARS-CoV-2	H1N1 ↓	↓ (0.99 *p* = 0.002)	↑ (−0.98 *p* = 0.001)	↓ (0.99 *p* = 0.001)
H1N1 3 h AdV2	None	Nd	Nd	Nd
SARS-CoV-2 3 h H1N1	None	Nd	Nd	Nd
SARS-CoV-2 3 h AdV2	SARS-CoV-2↓	↑ (−0.94 *p* = 0.05)	↑ (−0.95 *p* = 0.04)	↓
AdV2 3 h H1N1	AdV2 ↓	↓	None	↓ (0.99 *p* = 0.001)
AdV2 3 h SARS-CoV-2	AdV2 ↓	↓ (0.99 *p* = 0.003)	↓ (0.99 *p* = 0.001)	↓ (0.99 *p* = 0.001)
Superinfection at 24 h				
H1N1 24 h SARS-CoV-2	H1N1 ↑	↓ (−0.99 *p* = 0.008)	↓ (−0.99 *p* = 0.005)	↓ (0.99 *p* = 0.008)
H1N1 24 h AdV2	H1N1 ↓	↑ (−0.99 *p* = 0.002)	↓ (0.99 *p* = 0.005)	↓ (0.99 *p* = 0.005)
SARS-CoV-2 24 h H1N1	SARS-CoV-2 ↓	↑ (−0.98 *p* = 0.01)	None	↓
SARS-CoV-2 24 h AdV2	SARS-CoV-2 ↓	↑	↑	↓
AdV2 24 h H1N1	AdV2 ↑	↑	↑ (0.99 *p* = 0.04)	↑
AdV2 24 h SARS-CoV-2	AdV2 ↑	↑ (0.97 *p* = 0.02)	↓ (−0.96 *p* = 0.003)	↑ (0.97 *p* = 0.02)

^a^ Viral growth change was evaluated as reduced or enhanced fold change obtained during co-infection or superinfection compared to the fold change observed in single infection as reported in Figure 1 and Figure 2. ↑, increased replication; ↓, reduced replication. ^b^ Cytokine expression change was evaluated as reduced or enhanced fold change obtained during co-infection or superinfection compared to the fold change observed in single infection as reported in Figure 3 and Figure 4. Nd, not determined. ↑, increased expression; ↓, reduced expression. Specific viral and cytokine change in the co-infection is shown in different colors (black and red). Significant Person correlation between viral growth and cytokine change is reported. Significant Person correlation between viral growth and cytokine change is reported.

## Data Availability

All data generated or analyzed during this study are included in this published article.
